# Mobile devices and wearable technology for measuring patient outcomes after surgery: a systematic review

**DOI:** 10.1038/s41746-021-00525-1

**Published:** 2021-11-12

**Authors:** Stephen R. Knight, Nathan Ng, Athanasios Tsanas, Kenneth Mclean, Claudia Pagliari, Ewen M. Harrison

**Affiliations:** 1 Surgical Informatics, Centre for Medical Informatics, Usher Institute, University of Edinburgh, Edinburgh, UK; 2 School of Medicine, University of Edinburgh, Edinburgh, United Kingdom; 3 Usher Institute, University of Edinburgh, Edinburgh, United Kingdom

**Keywords:** Rehabilitation, Outcomes research

## Abstract

Complications following surgery are common and frequently occur the following discharge. Mobile and wearable digital health interventions (DHI) provide an opportunity to monitor and support patients during their postoperative recovery. Lack of high-quality evidence is often cited as a barrier to DHI implementation. This review captures and appraises the current use, evidence base and reporting quality of mobile and wearable DHI following surgery. Keyword searches were performed within Embase, Cochrane Library, Web of Science and WHO Global Index Medicus databases, together with clinical trial registries and Google scholar. Studies involving patients undergoing any surgery requiring skin incision where postoperative outcomes were measured using a DHI following hospital discharge were included, with DHI defined as mobile and wireless technologies for health to improve health system efficiency and health outcomes. Methodological reporting quality was determined using the validated mobile health evidence reporting and assessment (mERA) guidelines. Bias was assessed using the Cochrane Collaboration tool for randomised studies or MINORS depending on study type. Overall, 6969 articles were screened, with 44 articles included. The majority (*n* = 34) described small prospective study designs, with a high risk of bias demonstrated. Reporting standards were suboptimal across all domains, particularly in relation to data security, prior patient engagement and cost analysis. Despite the potential of DHI to improve postoperative patient care, current progress is severely restricted by limitations in methodological reporting. There is an urgent need to improve reporting for DHI following surgery to identify patient benefit, promote reproducibility and encourage sustainability.

## Introduction

The worldwide use of surgical treatments is increasing, with approximately one in ten people undergoing a surgical procedure each year in high-income countries^1,2^. Following discharge, patients assume primary responsibility for monitoring their own recovery and differences in adhering with both this and related self-care recommendations, can produce variable outcomes. More than 10% of patients over 45 years old experience a major postoperative complication^3–5^, often following discharge^6^, which typically prompts readmission^7^ and is associated with increased postoperative mortality across a range of surgical populations^7,8^. However, even minor events following surgery, such as nausea and pain, are known to significantly affect patient satisfaction and wellbeing^9–13^.

Studies have already demonstrated that using digital health interventions (DHI) can help identify postoperative complications earlier, improve recovery, and provide safe follow-up which is acceptable to patients^10,14–18^. DHI, defined as ‘the use of mobile and wireless technologies for health to improve health system efficiency and health outcomes’^19^, provide the opportunity to connect patients and healthcare providers in real-time. For example, embedded sensors in mobile phones and wearable technology can capture data remotely, passively and continuously, providing opportunities to track physiological parameters and enable patients to self-report symptoms and signs, which can indicate their postoperative status. In surgery, DHI may include wearable activity trackers^20^, mobile phone applications^21^, real-time collection of patient-reported outcomes^22^ and/or multiple electronic devices forming a digital health kit^23^.

A growing body of literature evaluating DHI in surgery exists, including studies reporting its value in the assessment of postoperative recovery^24–26^ and its cost-effectiveness^27^. Meanwhile, the COVID-19 pandemic has accelerated the adoption of remote monitoring applications and use of digital health in all aspects of surgical workflow^22^. Medical professionals have increasingly utilised digital health interventions to monitor and review patients remotely^28^, encouraging resource expansion and potentially representing a paradigm shift in patient management^29^.

Previous systematic reviews reporting on digital health and surgery have focused on web-based interventions, where the use of mobile devices or real-time measurement of patient data was excluded^27,30,31^. In addition, the use of narrow inclusion criteria limit comparisons across the research field and hinder the identification of critical evidence gaps^19^. Despite the emergence of numerous DHI initiatives in surgery, there has been little discussion of the importance of rigorous reporting in this literature^30,31^.

We aimed to determine the current use, evidence base and reporting quality for mobile DHI in the postoperative period following surgery.

## Results

### Study characteristics

**Fig. 1:**
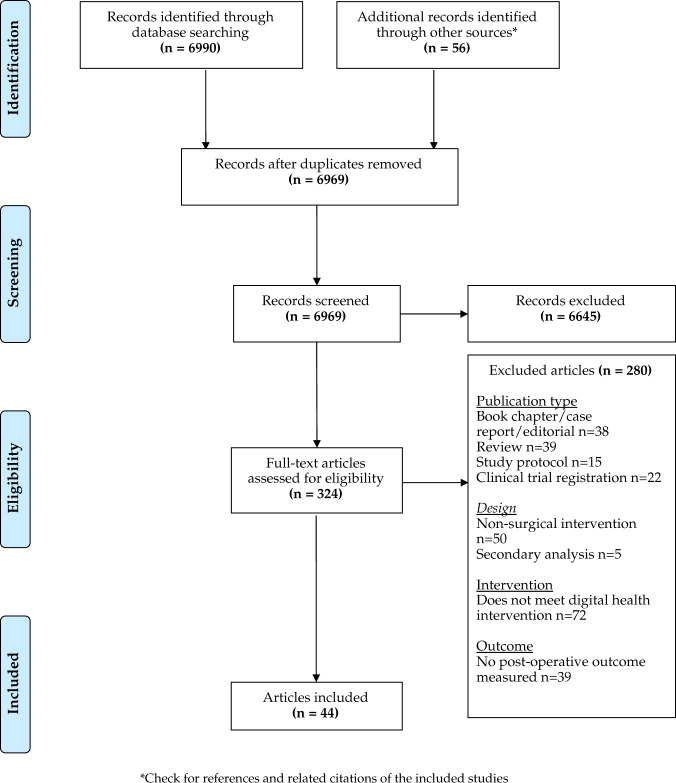
PRISMA diagram. Articles were published between January 2000 and May 2021, based on a search of Embase, Cochrane library, Web of Science, WHO Global Index Medicus, clinical trial registries and Google scholar databases (for details, see “Methods”).

Our review resulted in 324 full-text articles assessed for eligibility after initially screening 6969, with 44 articles (Fig. 1) ultimately included in this review^9,23,25,32–72^. Tables 1 and 2 provide descriptions of each study, recruiting 3890 patients in total across ten randomised controlled trials^9,32–40^, 17 prospective studies^25,42–54,71^ and 17 pilot or feasibility studies^23,56–70,72^.

**Table 1:** Summary of included randomised control trials.

Author	Procedures	Patient number	Digital health intervention (DHI)	Data collected	Control group	Length of intervention	Assessment of patient adherence	Measured patient adherence (%)
Mangieri et al., 2019	Laparoscopic sleeve gastrectomy	56	iPad mini with MyFitnessPal© application	Calorie counting & exercise tracking	Usual care	24 months	None	–
Campbell et al., 2019	Hip or knee replacement	159	SMS bot (StreaMD)	Pain and patient activity	Usual care	42 days	None	–
Hou et al., 2019	Lumbar spinal surgery	168	Mobile phone- based mHealth programme^a^	Guide and monitor patient rehabilitation	Usual care	90 days	Number of training sessions completed per week (arbitrary number)	65
Mousa et al., 2019	Infra-inguinal procedures	30	Tablet computer with an application (Enform)	Physiological parameters and QoL questionnaire	Usual care	30 days	None	–
Graetz et al., 2018	Gynaecological cancer surgery	29 (pilot)	Mobile application (Patient Care Monitor™)^a^	Postoperative symptoms. Automatic patient alerts using predetermined thresholds	Mobile app (no reminder)	30 days	Completion of all surveys	93
van der Meij et al., 2018	Laparoscopic abdominal procedures	344	Mobile application and activity tracker (UP MOVE, Jawbone)^a^	Postoperative recovery (PROMIS questionnaire) and daily step count	Usual care & placebo website	6 months	Completion of all questionnaires	87
Jaensson et al., 2017	Predominantly orthopaedic and general cases	997	Mobile application (RAPP)^a^	Postoperative recovery using SwQoR questionnaire	Usual care	14 days	None	–
Park et al., 2017	Total knee replacement	40	SMS messaging^a^	General health, pain, joint symptoms	Telephone consult	90 days	None	–
Armstrong et al, 2017	Breast reconstruction	65	Mobile application^a^	Wound images and pain scores. Red flags prompting in-person review	Usual care	30 days	None	–
Dabbs et al., 2016	Lung transplant	201	Mobile application (PocketPATH^®^)^a^	Self-monitored physiological parameters	Usual care	12 months	None	–

^a^Study inclusion criteria required the patient to own a mobile phone

**Table 2:** Summary of included prospective studies.

Author	Procedures	Patient n	Digital health intervention (DHI)	Data collected	Length of intervention	Assessment of patient adherence	Measured patient adherence (%)
Jonker et al., 2021	Oncological surgery	47	Mobile application^a^ (Self-Management system) and Fitbit Charge 2	Physical activity, temperature, blood pressure, weight, pain and symptoms	90 days	Completion of study follow-up assessment	79
Gräfitsch et al., 2020	Abdominal wall hernia repair	16	Santiago^®^ tablet, actimeter and pulse oximeter	Continuous activity levels; pain, oxygen saturation and wound images	30 days	Activity measurements available for the entire postoperative period	69
Panda et al., 2019	Soft tissue and abdominal	62	Mobile application (Beiwe)^a^	Continuous passive collection of raw smartphone accelerometer data	6 weeks	None	–
Carmichael et al., 2019	Inguinal hernia (most common), abdominal and thoracic procedures	175	Vivofit 3 (Garmin)	Mean daily step count calculated for each elective procedure type, including preoperative baseline	30 days	At least 2 weeks postoperative data available	68
Thijs et al., 2019	CABG	22	Fitbit Charge HR	Weekly average step count data downloaded at end of the study period	14 days	Accelerometer worn for the entirety of postoperative study period	77
Cole et al., 2019	Transsphenoidal surgery	7	Wristband device (Wavelet Health)	Multiple physiological parameters tracked (step count, calories, distance, heart rate, RR and sats)	Up to 13 days (average 8 days)	Transfer of data from device to cloud storage system	84
Argent et al., 2019	Total knee replacement	15	Shimmer3 inertial measurement unit	Accelerometer data used to guide and provide feedback on rehabilitation exercises	14 days	None	–
Scheper et al., 2019	Joint arthroplasty	69	Mobile application^a^	Wound symptoms and images	30 days	Use of application until day 30	59
Khanwalkar et al., 2018	Sinus surgery	288	Mobile application^a^	Pain and PROMIS pain interference	14 days	Completion of follow-up survey	89
Felbaum et al., 2018	Spinal surgery	56	Mobile application (TrackMyRecovery^®^)^a^	Patient education, pain scores and wound images	30 days	Downloaded and sent data through app	96
Anthony et al., 2018	Hand surgery	47	Text messaging via software robot^a^	Patient-reported pain and opiate use through daily automated text messages	7 days	Completion of all questionnaires	88
Gunter et al., 2018	Lower limb vascular surgery	40	Mobile application (WoundCheck)	Participant satisfaction and wound status (via app)	14 days	Daily submission of data	45
Ghomrawi et al., 2018	Range of elective paediatric surgical procedures	60	Actigraph wearable accelerometer	Time spent in grades of physical activity (light to intense). Data acquired at end of the study period	14 days	Wear accelerometer for at least 10 h each day of the study period	42
Pozza et al., 2018	Cosmetic surgery	57	Mobile messaging (SMS and MMS)^a^	Text message and wound images	7 days	Completed postoperative survey	91
Agarwal et al., 2018	Robotic laparoscopic prostatectomy	46	Fitbit Charge HR and mobile application^a^	Pre- and postoperative physical activity (measured by average step count)	Up to 15 days	None	–
Scott et al., 2017	Colorectal surgery	20	Mobile application (mHealth app from Seamless Mobile Health)^a^	Daily postoperative symptom tracker using pre-developed algorithm	14 days	Completed follow-up	85
Symer et al., 2017	Open and laparoscopic abdominal surgery	31	FitBit Charge HR and mobile application^a^	Daily symptom questionnaire and wound images. Automated alerts via app	30 days	Completed at least one app-related task ≥70% of the time	84
Sosa et al., 2017	Head and neck cancer resection	23	Mobile messaging (SMS and MMS)^a^	Text messages and wound images (on the SenseHealth app platform)	7 days	None	–
Castillo et al., 2017	C-section	105	Mobile application (how2trak)^a^	Wound images and surgical site infection symptoms	30 days	Submission of wound images up to 30 days	45
Higgins et al., 2017	ACL reconstruction	32	Mobile application (web-based)	Mobile app collecting pain scores, QoL (QoR-9) and wound images	6 weeks	None	–
Chiang et al., 2017	Total knee replacement	18	Accelerometer (brand not stated)	Accelerometer used periodically to measure the range of postoperative activity	6 weeks	None	–
Sun et al., 2017	Major gastrointestinal resection	20	VivoFit2	Daily steps are continuously collected using a secure group account	14 days	Wore device for at least 1 week after discharge	83
Abraham et al., 2017	Breast reconstruction	4	Smartwatch (Microsoft Band 2)^a^	Step count and physiological parameters streamed continuously via Wi-Fi	28 days	Daily collection of data	50
Carrier et al., 2016	Major colorectal resections	111	Mobile messaging^a^	Pain and postoperative symptoms captured using text messaging	7 days	Reply to all messages	90
Toogood et al., 2016	Total hip arthroplasty	33	Fitbit and mobile phone	Daily step count used as marker of patient activity	30 days	Transmit data for seven consecutive days	89
Tenhagen et al., 2016	Gastric sleeve or bypass	14	Internet-enabled weighing scales	Patients requested to weigh themselves at least once a week	1 year	Provided weight for ≥80% weeks	50
Debono et al., 2016	Lumbar discectomy	60	Mobile application^a^	Predetermined patient responses for pain and postoperative symptoms triggered response alarm	16 days	None	–
Mobbs et al., 2016	Lumbar spine surgery	30	FitBit zip	Average daily activity over each month. Data accessed through shared patient-investigator login	90 days	Accelerometer worn for an entire study period	93
McElroy et al., 2016	Cardiac surgery	27	Bluetooth-enabled tablet	Tablet linked to digital health kit (pulse oximeter, heart rate blood pressure cuff and weight scales)	30 days	None	–
Semple et al., 2015	Breast reconstruction and ACL repair	65	Mobile application	Postoperative pain, QoL (QoR-9) and wound photographs	30 days	Upload of at least one wound photograph each day	71
Dawes et al., 2015	Any colorectal procedure	20	Pre-programmed tablet computer	Postoperative health status survey completed daily	14 days	None	–
Palombo et al., 2009	Carotid endarterectomy	36	UMTS technology internet-linked video phone	Surgical wound, blood pressure and heart rate monitored every 4 h for 2 days	2 days	None	–
Martinez-Ramos et al., 2009	Range of ambulatory procedures	96	GPRS phone-based system	Wound images	14 days	None	–
Perez et al., 2006	Predominantly orthopaedic procedures	49	Mobile application(symbian OS phone)	Portable saturations probe readings and wound images	Not stated	None	–

^a^Study inclusion criteria required the patient to own a mobile phone

More than half of the studies were conducted in the United States (*n* = 24; 1556 patients)^23,32,33,35,36,39–42,44–50,52,56,59–61,65–67^, with only one originating from a low- or middle-income setting^34^. Orthopaedic procedures were represented in a quarter of studies (*n* = 10; 611 patients)^25,33,34,38,46,52,57,58,63,64^, with interventions taking place predominantly within the first 30 postoperative days^9,23,34,36,39,42–52,54–62,65–69,72^. Real-time data collection and autonomous delivery to clinicians for immediate review occurred in 31 studies^9,23,32,34,36,37,39,40,44–48,50–56,58,60–63,65–69,71^.

### Mobile phone-based interventions

Thirty one of the eligible studies used a mixture of mobile phone-based interventions^9,32–34,36–41,44–52,54–56,58,60–63,68–71^, with 20 using smartphone applications^9,32,36,37,39–41,44,45,47–50,54,55,58,62,63,70,71^. Remote assessment of wound images taken by the patient and evaluation of symptoms reported using validated tools were the most frequent aims of the mobile phone-based interventions^39,45,47,49,50,55,58,60,62,63,68–70^. In total, 19 individual mobile applications were described (Table 3). Only three of these were publicly available to download from either Android or Apple platforms^32,41,48^, while it was unclear what platform the others used. One application was available as a demonstration version, however, patient data entry was restricted^62^. Five studies used predetermined thresholds or algorithms to generate clinician alerts from patient responses^36,39,48,49,54^.

**Table 3:** Studies using mobile applications.

Author	Patient number	Surgical speciality	Study design	Mobile application	Industry or commercial interest	Platform	Purpose	Availability
Jonker et al., 2021	47	Oncologic surgery	Prospective	Self-management system (SMS)	Yes	Android	Activity monitoring, observations and postoperative symptoms	Not available
Panda et al., 2019	62	Soft tissue and abdominal	Prospective	Mobile application (Beiwe)	No	Android, iOS	Continuous passive collection of accelerometer data	Android and iOS
Mangieri et al., 2019	56	Bariatric surgery	RCT	MyFitnessPal©	Yes	Android, iOS	Encourage patient activity and weight loss	Android and iOS
Scheper et al., 2019	69	Orthopaedics	Prospective	Innovattic	Yes	Not stated	Symptom tracker and uploading of wound images	Not available
Graetz et al., 2018	29	Obstetrics and gynaecology	RCT	Adapted version of Patient Care Monitor^™^	Yes	Web-based	Records postoperative symptoms	Not available
van der Meij et al., 2018	344	Gastrointestinal surgery	RCT	Unnamed		Web-based	Provided information on recovery and tracked recovery	Not available
Khanwalkar et al., 2018	288	ENT surgery	Prospective	Unnamed		Not stated	Measures PROMs	Not available
Felbaum et al., 2018	56	Neurosurgery	Prospective	TrackMyRecovery^®^		Android, iOS	Sends reminders, measures pain scores and wound images	Not available
Gunter et al, 2018	40	Vascular surgery	Prospective	WoundCheck		iOS	Uploading of wound images and recovery progress	Not available
Jaensson et al., 2017	997	Day surgery	RCT	RAPP		Not stated	Assesses postoperative recovery	Not available
Armstrong et al., 2017	65	Breast surgery	RCT	Unnamed		Not stated	Wound images, pain and QoL	Not available
Scott et al., 2017	20	Colorectal surgery	Prospective	Seamless mobile health	Yes	Android, iOS, Blackberry OS	Symptom tracker	Android and iOS
Symer et al., 2017	31	Gastrointestinal surgery	Prospective	Unnamed		Android, iOS	Symptom tracker and uploading of wound images	Not available
Sosa et al., 2017	23	Head and Neck	Prospective	SenseHealth		Android, iOS	Symptom tracker and uploading of wound images	Not available
Castillo et al., 2017	105	Obstetric and gynaecology	Prospective	How2trak		Not stated	Symptom tracker and uploading of wound images	Android only (demonstration only)
Higgins et al., 2017	32	Orthopaedics	Prospective	QoC Health		Not stated	Symptom tracker measures recovery and uploading of wound images	Not available
Dabbs et al., 2016	201	Transplantation	RCT	PocketPATH^®^		Not stated	Records daily health indicators	Not available
Debono et al., 2016	60	Neurosurgery	Prospective	SOVINTY e-Healthcare services software		Not stated	Records postoperative symptoms	Not available
Semple et al., 2015	65	Multiple specialties	Prospective	Unnamed		Not stated	Measures pain and recovery scores	Not available
Perez et al., 2006	49	Day surgery	Prospective	Unnamed		Symbian OS phones	Uploading of wound images	Not available

*ENT* ear, nose and throat surgery, *iOS* apple mobile device operating system, *QoL* quality of life, *PROMS* patient-reported outcome measures.

Twenty-one studies required patients to own a mobile device^9,34,36–41,44–46,48–51,54,58,60–62,66,71^ excluding up to a third of patients approached as a result^47,48^. Where participants were provided with a mobile device, participant age was higher (56.1 vs. 53.1 years), with only two studies explicitly recruiting older patients (≥60 years old)^52,71^.

Mobile phone-based interventions included multimodal patient feedback programmes^32,34,37^, postoperative recovery tracking^39,57^ and patient education^9^. These frequently reduced the requirement for postoperative in-person reviews and reduced inappropriate patient emergency department use^39,45,54,70^. Some interventions were demonstrated to encourage quicker postoperative recovery and reduce analgesic requirements^33,37,41,46^ while postoperative complications could be identified earlier through both mobile messaging and wound photographs^60,63^. However, complication rates were similar to control groups in all studies where reported (range 2.0–7.1%)^35,37^. In those studies utilising predefined algorithms and thresholds, none had been previously validated within another patient cohort^36,39,48,49,54^.

### Wearable devices

Accelerometer-based devices were the most commonly represented wearable device, measuring postoperative patient physical activity or intensity (*n* = 14) via FitBit^25,43,49,52,61,72^ smartwatch^42,65,66^ or other devices^37,56,59,64,71^. Eight studies required the synchronisation of wearable devices to a mobile phone, together with manual download by a clinician on study completion, to allow data analysis^25,42,43,49,57,59,64,72^. Studies using wearables for continuous patient monitoring were less common, with only three studies reporting the use of automated data feeds for real-time clinical analytics and feedback^49^^,54,66^.

Studies demonstrated that increases in step count postoperatively correlated with age^52,61^, body build^61^ and operative approach (open versus keyhole procedures)^43,52^. Accelerometer activity data also demonstrated postoperative complications could be identified at an earlier stage^42^, were associated with other physiological parameters^56^ and correlated with complication scores such as the Comprehensive Complication Index^65^. Activity recovery curves were also demonstrated for common abdominal and thoracic procedures^42^. Only one study utilised in-built smartphone accelerometers, which demonstrated postoperative complications reduced daily exertional activity compared to baseline up to 6 weeks after surgery^41^.

A single randomised trial^37^ used a wearable device as part of a multimodal intervention, however, only a proportion of patients received this device, as patients were required to own a compatible smartphone. The study’s authors did not report results based on device data, with a return to normal activity measured through the validated Patient-Reported Outcomes Measurement Information System^®^ (PROMIS) score.

### Measured outcomes

The majority of studies reported postoperative recovery as their main outcome (Table 4)^9,25,33,34,37,38,41–43,52,54,56,59,61,64–67,72^. Additional primary outcomes included the impact of DHI on pain management^33,34,44,46^, postoperative complications^50,51,58,60,68^, symptom monitoring^36,40^, surgical site infection^35,47,55,62,69,70^ and hospital resource use^23,35,39,45,63^. Two studies determined the ability of DHI to aid postoperative weight loss following bariatric surgery^32,53^, while four studies solely focused on determining the feasibility of a DHI in postoperative follow-up^48,49,57,71^.

**Table 4:** Outcomes measured across included studies.

Primary outcome	Author	Study design	Procedures	Patient number	Length of intervention	Main finding
Postoperative pain management	Campbell et al., 2019	RCT	Hip or knee replacement	159	42 days	Stopped taking narcotics 10 days sooner (*P* < 0.001)
Postoperative pain management	Hou et al., 2019	RCT	Lumbar spinal surgery	168	90 days	No difference in pain scores
Postoperative pain management	Khanwalkar et al., 2018	Prospective	Sinus surgery	288	14 days	Similar analgesic requirements across all included procedures
Postoperative pain management	Anthony et al., 2018	Prospective	Hand surgery	47	7 days	Pain trended down sequentially over the first week
Postoperative complications	Scheper et al., 2019	Prospective	Joint arthroplasty	69	30 days	80% patient-reported complications concorded with physician diagnosis.
Postoperative complications	Pozza et al., 2018	Prospective	Cosmetic surgery	57	7 days	All three complications were detected earlier in the postoperative period
Postoperative complications	Sosa et al., 2017	Prospective	Head and neck cancer resection	23	7 days	Patients with postoperative complications are more likely to use a platform (*P* < 0.001)
Postoperative complications	Carrier et al., 2016	Prospective	Major colorectal resections	111	7 days	Alerts led to early, timely detection of postoperative complications
Postoperative complications	Palombo et al., 2009	Prospective	Carotid endarterectomy	36	2 days	The intervention allowed safe early discharge in selected patients
Postoperative symptom monitoring	Graetz et al., 2018	RCT	Gynaecological cancer surgery	29 (pilot)	30 days	Feasible and acceptable to the patient population. Reminders increased use of a mobile application.
Postoperative symptom monitoring	Dabbs et al., 2016	RCT	Lung transplant	201	12 months	Self-monitoring increased with app use, with patients more likely to report critical indicators (OR 5.11; *P* < 0.001)
Postoperative recovery	Gräfitsch et al., 2020	Prospective	Abdominal wall hernia repair	16	30 days	60% of patients regained preoperative activity levels within 3 weeks
Postoperative recovery	Panda et al., 2019	Prospective	Cancer surgery	62	6 weeks	Patients with postoperative complications showed lower activity and ability to achieve 60 min of exertional activity
Postoperative recovery	Campbell et al., 2019	RCT	Hip or knee replacement	159	42 days	Patients in the intervention group exercised for longer (8.6 min per day; *P* < 0.001)
Postoperative recovery	Hou et al., 2019	RCT	Lumbar spinal surgery	168	90 days	Disability improved in mHealth group
Postoperative recovery	Carmichael et al., 2019	Prospective	Inguinal hernia (most common), abdominal and thoracic procedures	175	30 days	Recovery trajectories have the potential to predict postoperative complications up to 3 days before readmission
Postoperative recovery	Thijs et al., 2019	Prospective	CABG	22	14 days	Higher physical activity has seen following minimally invasive procedures
Postoperative recovery	Cole et al., 2019	Prospective	Transsphenoidal surgery	7	Up to 13 days (average 8 days)	Step count fell by 45% following surgery
Postoperative recovery	van der Meij et al., 2018	RCT	Laparoscopic abdominal procedures	344	6 months	Five-day reduction in return to normal activities (21 days vs. 26 days; *P* = 0.007)
Postoperative recovery	Ghomrawi et al., 2018	Prospective	Range of elective paediatric surgical procedures	60	14 days	Different activity curves demonstrated for patients undergoing in-patient and out-patient procedures
Postoperative recovery	Agarwal et al., 2018	Prospective	Robotic laparoscopic prostatectomy	46	Up to 15 days	Greatest reduction in postoperative step count seen in obese and men aged >65 years old
Postoperative recovery	Jaensson et al., 2017	RCT	Predominantly orthopaedic and general cases	997	14 days	Improved recovery in several symptom domains
Postoperative recovery	Park et al., 2017	RCT	Total knee replacement	40	90 days	SMS messages achieved similar postoperative recovery compared to routine care
Postoperative recovery	Chiang et al., 2017	Prospective	Total knee replacement	18	6 weeks	Postoperative range of motion improved if haemostatic agent used intra-operatively
Postoperative recovery	Sun et al., 2017	Prospective	Major gastrointestinal resection	20	14 days	Median step count at day 7 correlated with the Comprehensive Complication Index (CCI)
Postoperative recovery	Abraham et al., 2017	Prospective	Breast reconstruction	4	28 days	Variance in total sleep duration is a potential marker of recovery
Postoperative recovery	Toogood et al., 2016	Prospective	Total hip arthroplasty	33	30 days	Activity increased in a step-wise fashion post-discharge. Age and operative approach were associated with postoperative activity
Postoperative recovery	Debono et al., 2016	Prospective	Lumbar discectomy	60	16 days	Deviations in expected postoperative recovery were identified early, reducing emergency department admissions
Postoperative recovery	Mobbs et al., 2016	Prospective	Lumbar spine surgery	30	90 days	Daily mean step count and distance had improved at follow-up
Postoperative recovery	Dawes et al., 2015	Prospective	Any colorectal procedure	20	14 days	Patients felt more aware of the recovery process and connected with their surgical team
Surgical site infection	Mousa et al., 2019	RCT	Infra-inguinal procedures	30	30 days	No difference in 30-day surgical site infection rates
Surgical site infection	Gunter et al., 2018	Prospective	Lower limb vascular surgery	40	14 days	Surgical site infection correctly identified in 87% of cases
Surgical site infection	Castillo et al., 2017	Prospective	C-section	105	30 days	One surgical site infection identified through intervention
Surgical site infection	Semple et al., 2015	Prospective	Breast reconstruction and ACL repair	65	30 days	All wound complications were correctly identified
Surgical site infection	Martinez-Ramos et al., 2009	Prospective	Range of ambulatory procedures	96	14 days	Two-thirds of patients had their wound concerns successfully resolved without need for hospital review
Surgical site infection	Perez et al., 2006	Prospective	Predominantly orthopaedic procedures	49	Not stated	Images modified original treatment plans and avoided emergency department attendance for 88%
Follow-up requirements	Mousa et al., 2019	RCT	Infra-inguinal procedures	30	30 days	No difference in 30-day readmission rates
Follow-up requirements	Felbaum et al., 2018	Prospective	Spinal surgery	56	30 days	Mobile application reduced hospital visits
Follow-up requirements	Armstrong et al., 2017	RCT	Breast reconstruction	65	30 days	Fewer in-person follow-up care visits in mHealth group (0.4; *P* < 0.001)
Follow-up requirements	Higgins et al., 2017	Prospective	ACL reconstruction	32	6 weeks	Intervention reduced the need for routine follow-up
Follow-up requirements	McElroy et al., 2016	Prospective	Cardiac surgery	27	30 days	Readmissions similar between intervention and control groups
Weight loss	Mangieri et al., 2019	RCT	Laparoscopic sleeve gastrectomy	56	24 months	Application aided longer-term weight loss at 12 months post-surgery
Weight loss	Tenhagen et al., 2016	Prospective	Gastric sleeve or bypass	14	1 year	Excess weight loss >40% in all patients
Feasibility	Jonker et al., 2021	Prospective	Oncological procedures	47	90 days	Older patients (≥65 years old) can successfully perform home monitoring using DHIs, with good usability and acceptability
Feasibility	Argent et al., 2019	Prospective	Total knee replacement	15	14 days	Biofeedback system improved rehabilitation experience for patients
Feasibility	Scott et al., 2017	Prospective	Colorectal surgery	20	14 days	Low use of mobile application associated with inappropriate emergency department presentation in 63% of cases
Feasibility	Symer et al., 2017	Prospective	Open and laparoscopic abdominal surgery	31	30 days	Patients generated an average of 1.1 alerts, but 50% of patients struggled to upload photographs

Differences in study methodology and outcome definitions limit conclusions on the effectiveness of DHI across each outcome. However, DHI demonstrated a strong ability to track postoperative analgesic requirements^33,34,44,46^ and patient recovery^9,25,33,34,37,38,41–43,52,54,56,59,61,64–67,72^ while consistently reducing hospital resource use in the postoperative period^39,45,63,70^. The capture of longer-term outcomes were also possible beyond 30 days, particularly for orthopaedic procedures^25,34,38,63,64^ and to monitor weight loss^32,53^. DHI were also able to identify complications at an early stage^51,60^ and correctly classify wound infection in the majority of patients^47,55,62^, demonstrating good agreement with physicians^55,58^.

### Patient adherence

Twenty-five studies reported patient adherence with digital health interventions^25,34,36,37,42–49,51–53,55,56,58–60,62,65,66,72^ however this assessment varied widely (Tables 1 and 2). Patient adherence ranged between 42 to 96%, however, no included studies used a validated assessment method. Adherence was generally found to be highest within the first 2 weeks postoperatively^55,58,72^ with adherence falling for longer-term interventions^34,55,62^. Patients with complications were more likely to use DHI^50^, while limited use of mobile applications was associated with high rates of inappropriate emergency department presentation following major colorectal resection^48^. High patient satisfaction was reported in multiple studies^23,33,39,45,47,53,54,57,69,71^ however patients also found some DHI to be intrusive^36,53,58,71^ while none reported the carers’ use or experience of the intervention.

### Reporting quality and bias

**Fig. 2:**
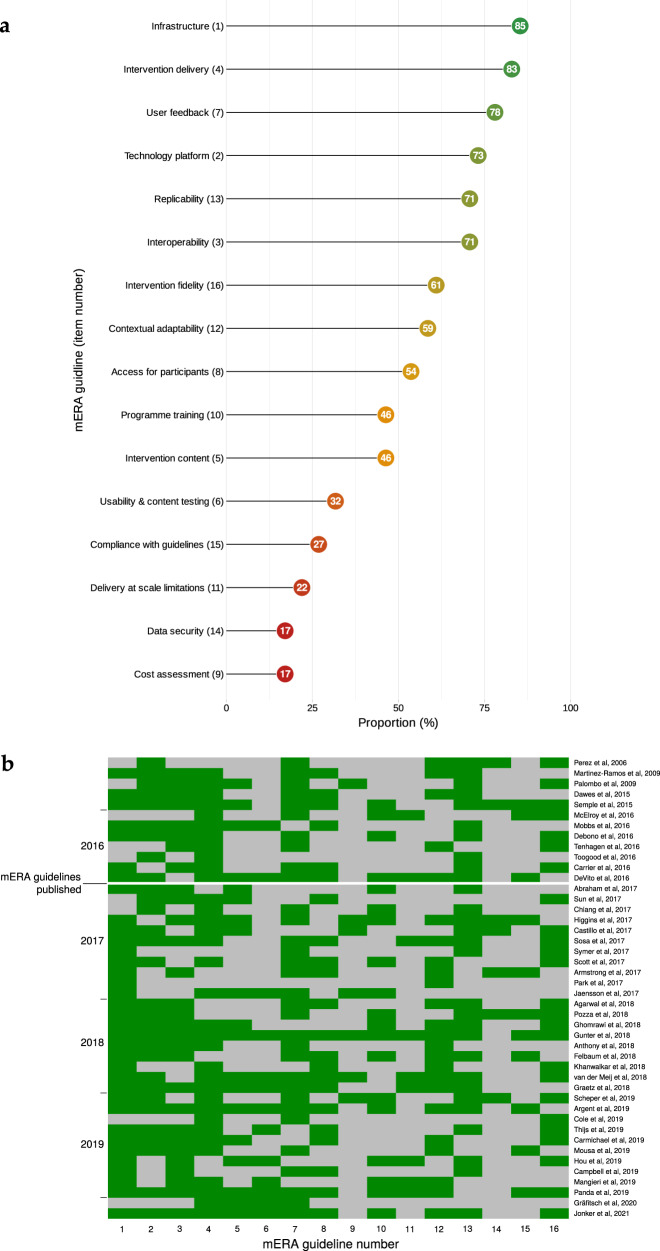
Reporting quality across included studies. Reporting quality for each mERA guideline domain (**a**) and temporal relationship (**b**). mERA guideline item number contained within parentheses.

Overall, reporting quality was suboptimal, particularly within the items of data security, cost assessment and patient engagement during intervention development (Fig. 2a). Only one domain, the presentation of infrastructure availability to support technology within the study location (item 1), was consistently reported across all studies. Other domains, including data security, cost assessment and scalability; were frequently under-reported, demonstrating poor standardisation and limiting comparability across studies. The median score was 8 (range 2 to 15), while only nine (19%) studies scored above 10^36,37,40,41,47,55,57,63,71^ No obvious trends in reporting quality were detected over time, despite the publication of a mobile health evidence reporting and assessment (mERA) and World Health Organisation Monitoring and evaluating digital health interventions in 2016 (Fig. 2b). No association was found between study design, device and quality score.

**Fig. 3:**
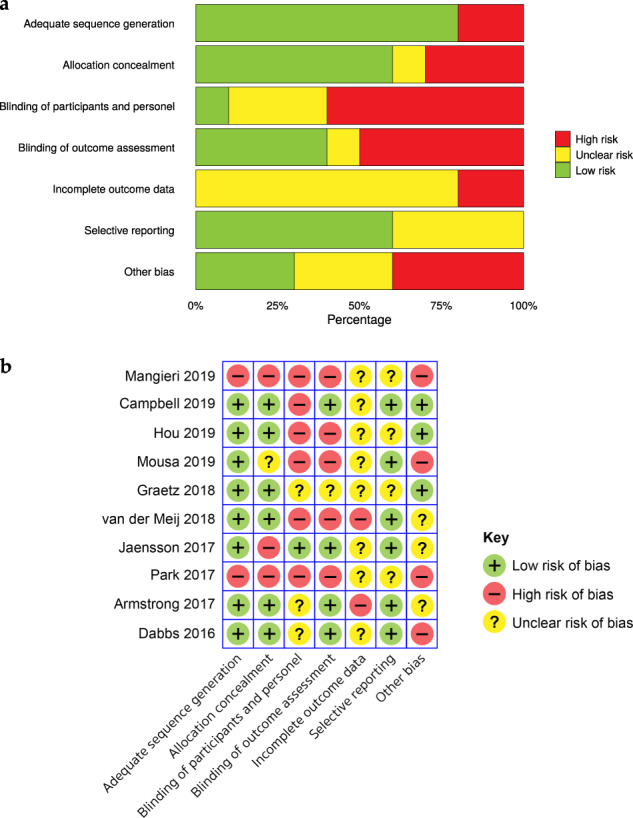
Risk of bias assessment. Overall summary (**a**) and individual bias assessment (**b**) for included randomised controlled trials assessed using the Cochrane collaboration tool.

Critical appraisal revealed that all the eligible randomised studies had a high risk of bias in at least one defined outcome, primarily at the allocation and blinding stages (Fig. 3). Prospective studies also showed a high risk of bias, demonstrated during blinding and recruitment of consecutive patients (Supplementary Table 1). Furthermore, only two studies included a control group^23,68^ and only one performed a sample size calculation a priori^56^.

## Discussion

To our knowledge, this is the first systematic review to have investigated the use and effectiveness of mobile DHI in postsurgical care, including a rigorous assessment of current reporting quality. The increasing affordability and widespread use of mobile technologies presents new opportunities to remotely monitor patient-centred health metrics during the postoperative period. In this review, we evaluated the use of DHI to complement conventional postoperative care across 42 studies. The wide diversity in the types of patient population, intervention and outcome measures were reported, while methodological reporting was found to be suboptimal across multiple domains.

Overall, the results indicate that regular acquisition of objective wound data (from images), patient-reported outcome data (from validated self-report tools) or continuous activity data (from wearables) can improve the assessment of postoperative recovery^26^. Combining remote assessment with active clinical prompts or patient advice (whether via automated or manual checking) also has the potential to reduce complication rates. Randomised studies included in this review demonstrated that DHI may facilitate patient recovery following major operations^9,37^, reduce inappropriate service use^39,40^ and improve longer-term outcomes in bariatric surgery^32,33^. Despite these opportunities, our review revealed a number of issues with the existing evidence base which require to be addressed if this potential is to be fulfilled.

DHI can provide an opportunity for patient engagement, support and self-care^73,74^, providing a bridge between clinical services and patients’ homes and helping to mitigate social isolation paving new ways to explore two-way interactions. Despite these opportunities, the research studies reviewed herein captured in this review made little reference to engaging patients in the development of the DHI and only one study was designed to engage patients in their care or in reviewing their own data^37^. Given the critical role of clinician-patient partnerships in the successful delivery of interventions and in supporting shared care, this seems like a missed opportunity and we would encourage future patient-centred research and interventions^73^. Many of the studies reported high levels of exclusion amongst patients who did not possess the relevant mobile technology, suggesting that more work on inclusive design is needed to avoid exacerbating the ‘digital health divide’^75^. The case for better patient engagement, or carers supporting an individual’s recovery, may also mitigate the well-known problem of patient attrition from digital health interventions^76^.

Published studies on the use of DHI in surgical populations came almost exclusively from high-income countries, particularly the USA. This is likely reflects both the research funding environment in different regions and the lack of financial accessibility of smartphones and wearables in resource-constrained countries. However, the often significant distance patients travel for surgical care in low- and middle-income countries, combined with difficulties in determining early outcomes in these settings^77^, offers huge potential for postoperative patient outcome reporting and is a legitimate candidate for global health research funding^26^.

Aggregated day level summaries of patient activity were commonly reported, with few exploring the potential of other accelerometer metrics to predict postoperative complications, such as sleep quality^78,79^ or activity intensity^26,80^. Wearable devices were found to generally associate well with operative characteristics and complication severity, however considerable variability within patient cohorts existed, highlighting the need to be developing more personalised models^42,56,65,81^ Large error values originating from manufacturers’ algorithms^82,83^, lack of standardised procedures for optimising accuracy^82^ and small patient cohorts may explain this variance. Data were also frequently only available to clinicians for ‘offline’ analysis upon study completion, demonstrating the current limited ability of accelerometer technology to assist management of a larger population through preloaded signal analysis algorithms and timely clinical review^84^.

Companies often have a market strategy that relies on proprietary algorithms and closed data sets, making it difficult to evaluate these innovations. This problem is exacerbated when such algorithms are updated, complicating longitudinal comparisons of measures even within the same brand device. We recommend further research investment in Open Software and the sharing of appropriately anonymised datasets for meta-analysis, to encourage sustainable and trustworthy innovations of this type. This is particularly important as we move towards more automated methods involving artificial intelligence, where the ability to scrutinise algorithmic decision making will become increasingly crucial for patient safety and clinical accountability^84^.

Methodological reporting across the included studies was of variable quality. Current reporting inconsistency is problematic, limiting researchers’ and policy makers’ ability to understand programme details and determine the impact on health systems^85^. Moreover, continued suboptimal reporting will limit future comparison and study reproducibility. The lack of data security information is particularly concerning and in contrast to the high priority given to security and privacy in electronic health records in general^55,86,87^. Patients identify security as the single most important barrier to technology use postoperatively^15^ and future public confidence in DHI may be eroded if patient confidentiality is felt to be at-risk^88,89^.

Patient adherence reporting is a key component of the mERA guidelines to determine patient engagement, user interaction and DHI fidelity. However, there was wide variation in the definition and assessment of patient adherence within included studies, which restricted more detailed comparison. This suggests the development and validation of a standardised tool, detailing specific metrics on how patient adherence should be defined in DHI studies is needed.

Furthermore, cost assessment was also limited, with basic information on financial costs to design and develop DHI from the perspective of all end-users omitted. Digital health is often assumed to be cost-effective^27^, however a lack of evidence to substantiate this remains a barrier to implementation and policy investment^90^. Insufficient detail prevents meaningful comparison with existing care, while the cost of adoption in postoperative surgical settings cannot currently be justified without assessment relative to meaningful clinical outcomes^91^.

Despite widespread publication and being extensively accessed^19,85,92^ mERA guidelines were poorly represented within included studies. Designed to address the gaps in comprehensiveness and quality of reporting on the effectiveness of digital health programmes, by an expert committee convened by the World Health Organization (WHO), implementation of all items should be achievable across all income strata. We found no evidence of temporal change in reporting quality, with our findings demonstrating urgent action is required to achieve consistent and comprehensive reporting of digital health interventions. Therefore, we strongly recommend journal editors make mERA checklist completion a mandatory condition for acceptance, similar to other reporting guidelines^93–95^.

Some limitations should be highlighted. As our search was only limited to the English language, we may have excluded relevant publications if they were not published in English. In addition, the omission of studies originating from low and middle-income countries is possible, with underreporting of DHI known to occur in studies outside the United States or without an industry sponsor^96^. Due to the heterogeneity of included studies and the quality of methodological reporting, we were unable to answer how DHI can impact specific clinical outcomes. Therefore, reported findings should be cautiously interpreted towards the current assessment of how digital health can improve patient outcomes following surgery until additional, higher-quality studies are available.

DHI to monitor postoperative recovery has been used across a broad range of surgical specialities, particularly within the United States. Devices are generally acceptable to patients and have been shown to identify postoperative complications early. Current studies report findings on small cohorts, infrequently engage patients during the design or delivery of the intervention and utilise patient-generated data in a passive manner. The requirement to own a mobile device considerably limits patient inclusion, while urgent improvements in the reporting of data security and cost-effectiveness is needed.

In order to advocate for the widespread use of digital health in the monitoring of postoperative patient recovery, additional high-quality research is needed prior to integration into the healthcare environment. Particular attention to reporting quality is advised, to ensure these studies can be replicated and provide the opportunity for equitable comparison.

## Methods

### Design

An electronic systematic search of Embase, Cochrane Library, Web of Science, WHO Global Index Medicus, clinical trial registries and Google scholar databases in accordance with the PRISMA guidelines was performed^93^. The PROSPERO international systematic review registry^97^ was searched to ensure a similar review had not previously been performed and the protocol was registered accordingly (CRD42019138736).

A thorough search was undertaken using the following Medical Subject Heading (MeSH) terms: ‘cellular phone’; ‘microcomputers’; ‘smartphone’, ‘iphone’; 'android’; ‘mobile’; ‘ipad’; ‘tablet’; ‘text message’; ‘sms’; ‘e-health’; ‘telemedicine’; ‘digital health’; ‘wearable’; ‘mobile health’; ‘mHealth’; and ‘surgery’; ‘postoperative’. The search was structured to ensure variations such as capitalisation, plurals and alternative phrases were captured (Supplementary Information 1). Search limits applied were English language, full-text, humans and articles published from 2000 (last search 18 May 2021). Case reports and editorials were excluded, with conference abstracts and reviews screened to assist in identifying related full-text articles prior to exclusion.

The title and abstract of all identified articles were screened independently by two authors (S.R.K. and N.N.), with those meeting the inclusion criteria screened further by full-text review. Any disagreements were resolved by discussion to reach a consensus. Reference lists of relevant articles were reviewed, together with a search of grey literature and the National Clinical Trials Register (clinicaltrials.gov) to identify any further studies for inclusion.

Studies involving patients undergoing any surgery requiring skin incision where postoperative outcomes were measured using a DHI following hospital discharge were included. DHI were defined according to the mobile health evidence reporting and assessment (mERA) guidelines; 'the use of mobile and wireless technologies for health to improve health system efficiency and health outcomes'^19^, with web-based interventions excluded if stationary devices, such as a desktop computer, were only used^27^. The more generic term ‘digital health’ was selected to ensure all potential approaches, including mhealth, were systematically captured within this review^98^. Interventions containing only teleconsultation or patient education components were excluded due to the number of previously published reviews in this area^27,30,31^.

### Data extraction

Data were extracted using a standardised proforma (Supplementary Information 2), with partial duplication to ensure consistency. Included studies were evaluated for study design, participant number, participant characteristics, DHI and origin, study duration and main findings. The method used to assess patient adherence was also extracted and reported based on the original study authors’ criteria. A wearable device was defined as a small computing device containing a sensor worn somewhere on the body^99^.

### Quality assessment

Reporting quality was analysed using the validated mERA 16-item core checklist, which systematically assesses transparency and completeness in digital health studies^19^. All included publications and associated study protocols were reviewed independently for potential risk of bias by two authors (S.R.K. and N.N.), using the Cochrane Collaboration tools for randomised studies^100^ and the methodological index for non-randomised studies (MINORS)^101^, with the global ideal score varying between non-comparative (16) and comparative studies (24).

We aimed to determine the current use, evidence base and reporting quality for mobile DHI in the postoperative period following surgery.

### Reporting summary

Further information on research design is available in the Nature Research Reporting Summary linked to this article.

## Supplementary information

Supplementary Information

Reporting Summary

## Data Availability

No new or unpublished data is included within the study and all data is freely available.

## Code availability

All code relating to summary figure development is available on request to the corresponding author.
