# Supplementary material for: Mobile devices and wearable technology for measuring patient outcomes after surgery: a systematic review
Source: NPJ Digit Med. 2021 Nov 12;4:157. doi: 10.1038/s41746-021-00525-1 (PMC8590052; doi:10.1038/s41746-021-00525-1)
Supplement: Supplementary file 1 — Supplementary Information [file 41746_2021_525_MOESM1_ESM.pdf]

Supplementary Table 1. Search terms

EMBASE

1. exp cellular phone/
2. exp microcomputers/
3. (smart phone$ or smartphone$ or iphone$ or android or blackberry$).tw.
4. ((handheld or portable or mobile) adj2 (phone$ or device$)).tw.
5. (personal digital assistant$ or pda).tw.
6. (ipad$ or tablet$).tw.
7. text messag$.tw.
8. sms.tw.
9. (e-health or telemedicine or digital health or wearable* or mobile health or mHealth or digital health or eHealth or LORAWaN).ti,ab.
10. or/1-9
11. (surgery or surg*).ti,ab.
12. (oper$ or post$).ti,ab.
13. 11 and 12
14. 10 and 13
15. limit 14 to (human and english language and yr="2000 - Current")
16. limit 15 to (article or article in press or "review")

The Cochrane Library

#1 MeSH descriptor: [Cellular Phone] explode all trees
#2 MeSH descriptor: [Microcomputers] explode all trees
#3 ("smart phone*" or smartphone* or iphone* or android or blackberry*):ti,ab
#4 ((handheld or portable or mobile) near/2 (phone* or device*)):ti,ab
#5 ("personal digital assistant*" or pda):ti,ab
#6 (ipad* or tablet*):ti,ab
#7 "text messag*":ti,ab
#8 sms:ti,ab
#9 (e-health or telemedicine or digital health or wearable* or "mobile health" or mHealth or "digital health" or eHealth or LORAWaN):ti,ab
#10 #1 or #2 or #3 or #4 or #5 or #6 or #7 or #8 or #9
#11 surgery or surg*:ti,ab
#12 (oper* or post* or outcome* or peri$ or "peri-operative period"):ti,ab
#13 #11 and #12
#14 #10 and #13

Clinicaltrials.gov

mHealth AND surgery AND completed studies
mobile AND surgery AND completed studies
phone AND surgery AND completed studies
pda AND surgery AND completed studies
text AND surgery AND completed studies
txt AND surgery AND completed studies
ipad AND surgery AND completed studies

WHO International Clinical Trials Registry Platform

mHealth or mobile or phone or pda or text or txt or ipad and surgery in Intervention

Supplementary Table 2. Data extraction proforma

	SK	NN
Author		
Year of publication		
Country		
Study type		
Patient number		
Surgical speciality		
Digital health intervention (DHI)		
Surgical procedure included		
Intervention commenced		
Length of study / intervention		
Control group used		
Primary outcome(s) measured		
Other outcome(s) measured		
Mean age of patient		
Main results		
Autonomous data collection		
Accelerometer used		
Reported patient adherence		
Notes / comments		
References screened		
